# Mapping the covariate-adjusted spatial effects of childhood anemia in Ethiopia using a semi-parametric additive model

**DOI:** 10.3389/fped.2025.1559140

**Published:** 2025-08-21

**Authors:** Seyifemickael Amare Yilema, Yegnanew A. Shiferaw, Najmeh Nakhaeirad, Ding-Geng Chen

**Affiliations:** ^1^ Department of Statistics, College of Natural and Computational Science, Debre Tabor University, Debre Tabor, Ethiopia; ^2^ Department of Statistics, University of Pretoria, Pretoria, South Africa; ^3^ Department of Statistics, University of Johannesburg, Johannesburg, South Africa; ^4^ College of Health Solution, Arizona State University, Phoenix, AZ, United States

**Keywords:** geolocations, semi-parametric, spatial, anemia, Ethiopia

## Abstract

**Background:**

Globally, anemia poses a serious health challenge for children under the age of five, and Ethiopia is one of the countries significantly affected by this issue. The 2016 Ethiopian Demographic and Health Survey (DHS) data sets were employed to evaluate anemia risk among children aged 6–59 months. Due to limited research has been conducted on childhood anemia spatial disparities at the Ethiopian zonal level, and it is essential for developing zonal-level interventions for inform policy recommendations.

**Methods:**

This study was examined the geospatial disparities in anemia prevalence among children aged 6–59 months. We used a semi-parametric additive model with spatial smoothing to assess zone-level variation in anemia risk while adjusting for key covariates. Each predictor variable was spatially adjusted using non-parametric smoothing techniques based on geolocation parameters, and corresponding maps for each predictor.

**Results:**

A regularized random forest techniques was employed to identify the most influential predictors of childhood anemia and enhance the model predictive performance. Our findings revealed that the regional states of Somalia, Afar, and Dire Dawa exhibit the highest risk levels for childhood anemia. Furthermore, the risk of anemia in children varies spatially across different zones in Ethiopia. The most prominent hotspots for childhood anemia were in the country's Northeastern, Eastern, and Southeastern regions. In contrast, the areas with the lowest risk were in Northwestern, Western, and Southwestern zones of Ethiopia.

**Conclusion:**

The significant spatial disparities in anemia risk across the administrative zones of Ethiopia, indicating that the distribution of each predictor variable is not uniform. These findings provide valuable insights for policymakers, enabling the development of geographically targeted interventions to mitigate anemia risk at the zonal level.

## 1 Introduction

Anemia is a medical condition defined by a decrease in hemoglobin concentration or a reduction in red blood cell (RBC), falling below the normal range observed in healthy individuals. This condition presents a significant global health challenge, particularly among children. The consequences of anemia in children are significant with including growth retardation, compromised immune systems, and increased vulnerability to diseases, potentially leading to fatal outcomes (1–6). Furthermore, anemia harmfully affects mental, physical, and language development and scholastic performance (1–7). In female, the long-term implications of anemia include the risk of low birth weight infants and postpartum hemorrhage, particularly among low-income families (8, 9). In addition, anemia often associated with inadequate nutrition and poor overall health (8–10).

According to the World Health Organization (WHO) global report, 39.8% of under children five were anemic worldwide, with 60.2% of these cases occurring in Africa (9). In East Africa, the situation is even more concerning, as anemia have been affected more than 75% of children under five, with prevalence rates varying from 44% to 76% (11). In Ethiopia, the 2016 Demographic and Health Survey (EDHS) revealed that 57% of children under five were anemic, with regional differences ranging from 42% in the Amhara to 83% in the Somali regions (12). Furthermore, various studies conducted in different regions of Ethiopia have reported varying prevalence rates of anemia among children under five, research from Northeast Ethiopia, North Showa, the Tigray region, Hawassa Referral Hospital, and Gondar shows prevalence rates varying from 21.6% to 41.7% (13–17). In 2021, the Ethiopian government launched its second health sector transformation plan, aiming to significantly lower child mortality rates and address related complications, with the goal of fostering healthier communities by 2025 (18).

Mapping the spatial distributions of disease incidence and prevalence has long been a vital tool in spatial epidemiology research. Therefore, the methodology has been used in characterizing spatial patterns of risk, identifying public health risk factors, and predicting disease outcomes in diverse geographical contexts (19–22). In Ethiopia, some studies have explored in traditional spatial analysis and the identification of associated covariates at national and subnational levels (23–29). However, there is limited research examining how the non-parametric smoothing effects of each predictor variable relate to the spatial variations in children anemia risk across Ethiopian administrative zones.

This paper aims to map and visualize the covariate-adjusted spatial effects of childhood anemia prevalence in these zones, specifically focusing on children aged 6–59 months and employing robust statistical models, such as semiparametric additive models. Therefore, the novelty of this work has two folds over previous research. First, we explore the geographical differences in the prevalence of childhood anemia among the local administrations (zones) in Ethiopia. Second, our innovative approach utilizes individual-level data to map and visualize the covariate-adjusted spatial effects of childhood anemia in local Ethiopian administrative zones.

## 2 Methods and materials

### 2.1 Data sources

#### 2.1.1 Survey data sources

The 2016 Ethiopian Demographic and Health Surveys (DHS) provided the data used in this investigation. After fulfilling the requirements, these surveys were obtained from the DHS program website https://dhsprogram.com. The DHS employed a multistage sampling design. Enumeration areas (EAs), established during the 2007 Population and Housing Census, were randomly selected in the first stage. In the second stage, households were systematically chosen from the selected clusters. For the 2016 Ethiopian DHS, 10,641 households were randomly selected, averaging 28 households per EA from a total of 645 EAs, creating a nationally representative sample (30). Additionally, stratified sampling was utilized to consider residential status (rural vs. urban households).

#### 2.1.2 Spatial data sources

The DHS Program first made Georeferenced Global Positioning System (GPS) datasets publicly available in 2003. Individual records from DHS household surveys can be linked to these georeferenced datasets using unique survey identifiers. Since the early 2000s, recording GPS coordinates during surveys has become increasingly common. Over 120 surveys, including one conducted in Ethiopia, utilize GPS data.

To safeguard the privacy of respondents, the locations in these datasets undergo alterations through a process known as geo-masking or geo-scrambling (31). In this process, the latitude and longitude of survey clusters are relocated to new coordinates while adhering to specific guidelines: urban areas are displaced by 0–2 km, rural locations by 0–5 km, and 1% of the points (or every 100th point) are displaced by up to 10 km from their original locations.

**Figure 1:**
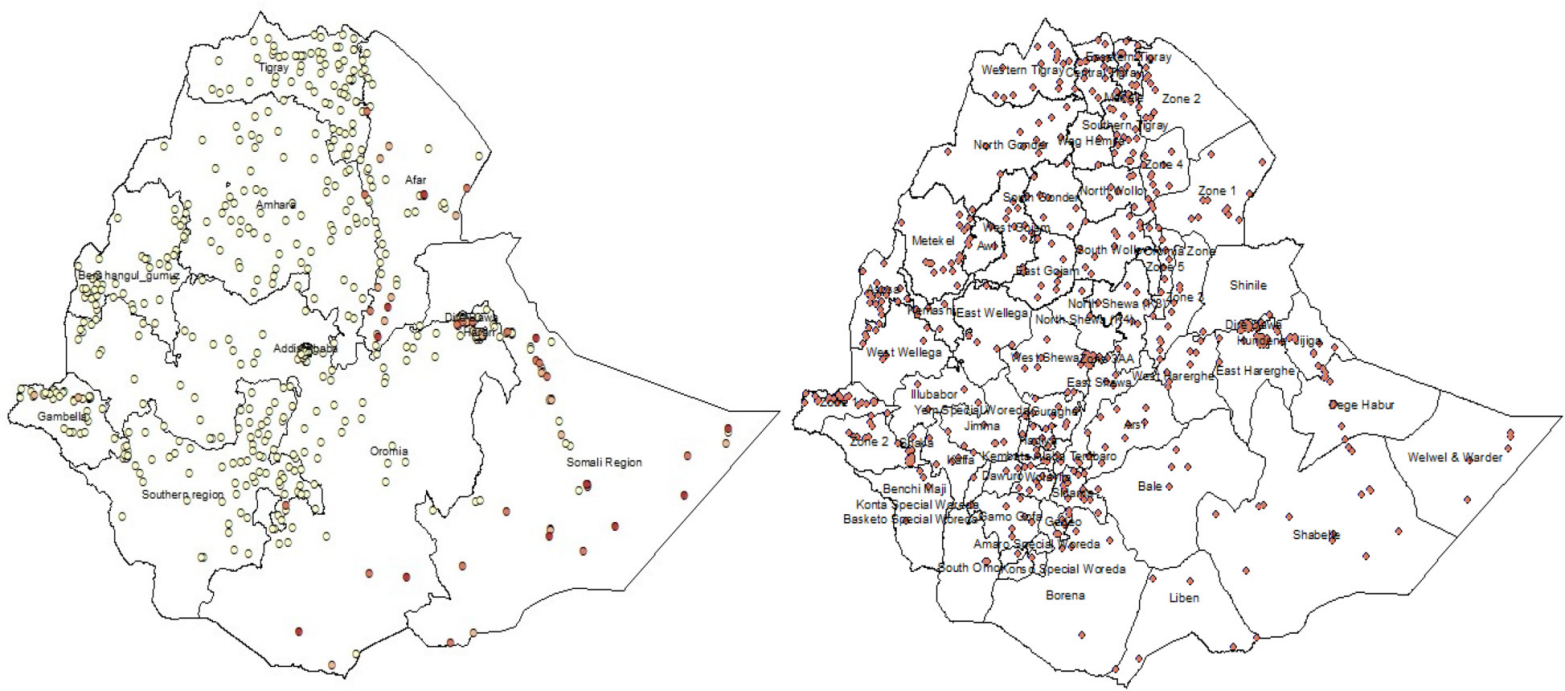
The EA datasets overlay across the regional and zonal maps of Ethiopia.

For more detailed information on the geographic displacement of DHS georeferenced data and the associated spatial variability, please refer to Spatial Analysis Reports 7–10 (31–33). The EA GPS datasets can be accessed from https://dhsprogram.com by submitting a reasonable request to the DHS program. Additionally, zonal shapefiles can be explored on the website https://www.diva-gis.org. Figure 1 illustrates the zonal and regional maps of Ethiopia, highlighting the anemia datasets within the EA.

### 2.2 Study variables

#### 2.2.1 Outcome variable: childhood anemia

Children aged 6–59 months who obtained consent from their parents, or another responsible guardian were subjected to anemia testing. Blood was drawn via a heel prick for children aged 6–11 months and from the palm side of the fingertip for those aged 12–59 months. Blood samples were collected using a hemoglobin HemoCue photometer, and the results were recorded immediately. In this study, the outcome variable, child anemia, is categorized as follows: a child's anemia is either present (yes = 1) or absent (no = 0). According to the World Health Organization's guidelines, children are considered anemic if their altitude-adjusted hemoglobin level is less than 11 g/dl (30).

#### 2.2.2 Independent variables (covariates)

**Figure 2:**
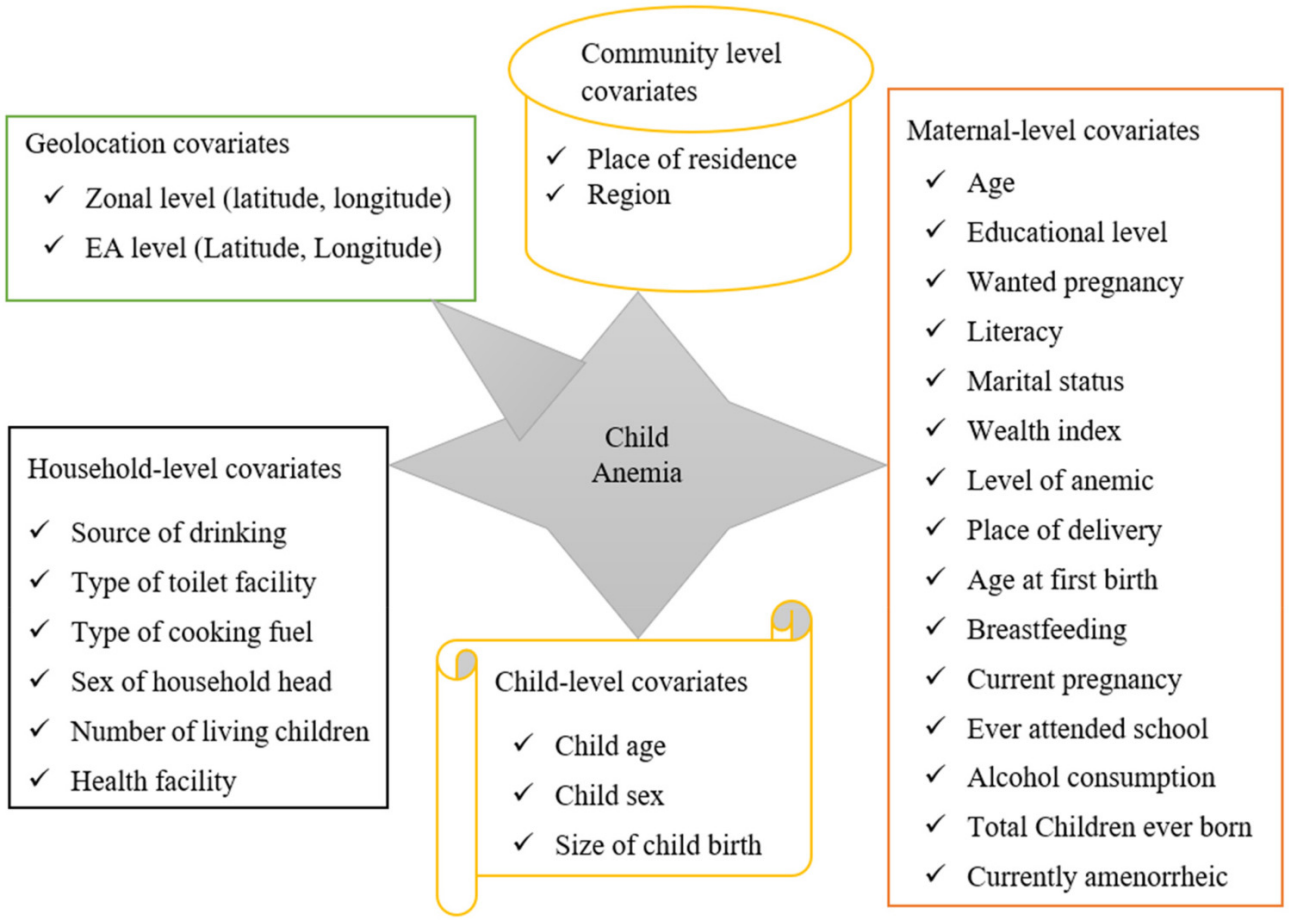
Conceptual framework for variable discerptions.

The potential variables for children under the age of five (6–59 months) are extracted from the 2016 DHS children's data to analyze the prevalence of anemia in children (30). These variables are selected using random forest feature importance methods. The entire dataset is examined with these methods, which effectively identify and eliminate unnecessary variables, thereby enhancing the model's predictive capability. By evaluating the relative significance of various variables within the dataset, this technique improves the effectiveness and performance of the statistical model. The variables included in this statistical analysis are illustrated in the framework below (Figure 2).

### 2.3 Inclusion and exclusion criteria

For the response variables, children aged 6–59 months who lived in the specified enumeration areas (community) met the inclusion requirements. Whereas children aged 6–59 months who did not have a hemoglobin test result were excluded. In addition, the covariates (independent variables) are selected based on literatures and the accessibility of the covariates in the DHS dataset. The candidate covariates for children under age 5 years (6–59 months) are obtained at different levels from the 2016 DHS kids' record for the analysis of child anemia prevalence. The potential candidate covariates displayed in Figure 2, are based on different literatures as follows: Child-level factors are age of child (in months), sex of child, size of child at birth, and pregnancy of the child wanted are chosen from these literatures (24, 34, 35). Maternal-level factors are age of mothers, mother's highest educational level, literacy, marital status of mothers, wealth index, mother's anemia level, place of delivery, maternal age at child birth, mother's breastfeeding, currently amenorrhea, currently abstaining from consuming alcohol, ever attended school, and mother's currently pregnancy (35, 36). Household-level factors are source of drinking water, type of toilet facility, type of cooking fuel, sex of household head, number of living children in the household (23, 25, 37). And finally community-level factors are place of residence, and region (35).

### 2.4 Importance variable selection

Variable importance is a method used in variable selection procedures. The regularized random forest variable importance algorithm is commonly used to identify the most important variables for machine learning and advanced statistical models (38). A regularized random forest model was employed to identify the most influential predictors of childhood anemia, as it effectively handles high-dimensional data and reduces overfitting by penalizing less important variables. Furthermore, it is resilient when dealing with nonlinear interactions and multicollinearity. This method made it possible to choose precisely which of the most significant predictors to incorporate into the geo additive model. The most significant variables typically emerge from the highest ranks of the predictor variable selections. This indicates each variable's statistical significance in relation to its impact on the constructed model. Furthermore, it ranks each independent variable based on its contribution to the model. Data scientists and statisticians utilize variable importance methods to filter out specific independent variables that provide value, rather than those that unnecessarily increase processing time (39–41).

In this study, we explored random forest variable importance methods to minimize the risk of leaving out useful variables while keeping a substantial contributing variable and eliminating the less contributing variables. We examined different literatures to identify the variables for this study (39, 42, 43), and on average, we observed that the retained variables typically had importance scores ranging from 5% to 30% of the maximum importance score. There are no widely accepted particular threshold hold values, despite the fact that these research offer insightful information (e.g., 5%–30% of the highest significance score) (41, 44). To keep a substantially contributing variable, the retaining variable significance score needs to be larger than 30% of the variable with the greatest important scores (>0.3×maximumimportancescore).

### 2.5 Geospatial statistical models

When the outcome of interest is binary, a generalized linear model (GLM) serves as a methodological framework for developing and applying models in disease mapping. GLMs are composed of random and systematic components that are interconnected through a link function. The probability distribution of the response variable *Y* is defined by the random component, which is assumed to belong to the exponential family and is characterized by a specific density function of the form given by

$$p_{Y}(y;\theta ;\phi )=\exp\{\frac{y\theta -b(\theta )}{a(\phi )}+c(y,\phi )\}$$

In the above density, θ is termed the canonical or natural parameter, c(.,.) the data function and the dispersion parameter, a(.) is a function of the dispersion parameter ϕ, and ϕ represents a nuisance parameter that characterizing the dispersion of response *Y*. Let us define the respondents of anemia risk for in *i*^th^ EA as

$$y_{ij}=\{\begin{array}{cc} 1, & \mathrm{if}\;j^{\mathrm{th}}\;\mathrm{child}\;\mathrm{in}\;\mathrm{the}\;\;i^{\mathrm{th}}\;\mathrm{EA}\;\mathrm{is}\;\mathrm{anemic}\\ 0, & \mathrm{otherwise} \end{array}$$

$y_{ij}$ follows a Bernoulli distribution with $P(y_{ij}=1)=\pi_{ij}$ being the probability that the anemia risk of the $j^{\mathrm{th}}$ child in the $i^{\mathrm{th}}$ geographical location is anemic and $P(y_{ij}=0)=1-\pi_{ij}$ is the probability that the anemia status of the $j^{\mathrm{th}}$ child in the $i^{\mathrm{th}}$ geographical location is non-anemic.

Estimating and mapping the spatial impacts of disease data at the individual level necessitate the application of various statistical models. Generalized additive models (GAMs), an extension of generalized linear models (GLMs), were first proposed by Hastie and Tibshirani (45). These models have become widely adopted for mapping point-based epidemiological data and are now regarded as indispensable tools in epidemiological analysis.

In the realm of geographical analysis, GAMs typically include smooth terms for spatial parameters (specifically, *x* and *y* coordinates) alongside a linear predictor for adjustment variables. Smooth terms effectively capture complex interactions, including those between the outcome and spatial elements, without imposing specific parametric forms on the relationships. GAMs provide a robust statistical framework for distributing illness risk across geographic areas by utilizing various covariates (46).

To encourage the use of GAMs for spatial disease mapping, the modeling of observations dispersed across a map with location coordinates $u_{i}$ and $v_{i}$ representing the location parameters for the $i^{\mathrm{th}}$ respondents i=1,2,….,N is taken into consideration. Let $Y_{i}$ denote the anemic outcome variables and $X_{i}$ represent the adjustment variables, which assumes that the anemia's distribution is a member of the exponential family. For a spatial effect analysis, the GAM can be defined as

$$g(\mu_{i})=\;\eta_{i}=\;\beta_{0}+x_{i}^{T}\beta +s(u_{i},\;v_{i}),\;i=1,\;2,\;\ldots ,\;N$$

where g(.) is the link function for the mean of the outcome $\mu_{i}=E(Y_{i})$ and the variance of the outcome is defined by the assumed probability model and denoted as $\mathrm{Var}(Y_{i})=V(\mu_{i},\;\phi )$ a function of the mean and nuisance parameter ϕ. The linear predictor by $\eta_{i}$, the spatial effects of interest are represented by the nonlinear smoothing function s $\left(u_{i},\;v_{i}\right)$ and β indicates a vector of coefficients associated with the adjustment covariate $x_{i}$ and finally $u_{i}$, and $\;v_{i}$ are the coordinates of longitude and latitude respectively. When fitting the model, the spatial effect model is decomposed into parametric and nonparametric parts:

$$f(u_{i},\;v_{i})=\;\gamma_{u}u_{i}+\gamma_{v}v_{i}+s(u_{i},v_{i}).$$

Therefore, the GAM model redefined as

$$g(\mu_{i})=\;\beta_{0}+x_{i}^{T}\beta +\gamma_{1}u_{i}+\gamma_{2}v_{i}+s(u_{i},v_{i}).$$

The parametric component of the spatial effect is fitted jointly with other adjustment variables using least squares, while the nonparametric term is fitted using a nonparametric smoother. A semi-parametric geo-additive model was applied to a variety of linear and nonlinear functional forms while accounting for spatial variability, making it ideal for assessing spatially structured on the anemia risk data. The semi-parametric geo-additive model has an advantage over parametric models in its functional flexibility, which improves accuracy and reduces biases, leading to more accurate estimates and findings. In addition, the semi-parametric geo-additive model offers a key benefit by allowing the simultaneous estimation of fixed, nonlinear, and spatial effects, thereby providing greater flexibility in capturing unobserved heterogeneity and spatial autocorrelation over the conventional GLMs.

A locally weighted scatterplot smoother (LOESS) is commonly used as the bivariate smoothing function for two geolocations, u and v, due to the significant variation in population densities across geographic areas (47–49). LOESS is particularly suitable because it retains the smooth characteristics of a kernel while adapting the size of the smoothing neighborhood to the local density. The smoothing parameter that defines the neighborhood can either be automatically selected by minimizing the Akaike Information Criterion (AIC) or the residual deviance (50). The AIC is commonly used for the optimal span size selection in this analysis for GAM model.

GAMs were used to smooth the predictor variables with a two-dimensional predictor (geolocation) and adjust linearly for confounding variables, resulting in a heatmap of odds ratios and other effect estimates (19). Permutation tests were performed for the null hypothesis to examine the relationship between the two-dimensional predictor and anemia status while controlling for adjusted covariates (13, 19). We set the default number of permutations to test the significance of the two-dimensional predictors as it saves calculation time.

Considering this, a *p*-value for testing the globally adjusted spatial effects is provided by the permutation test. The null distribution is represented by the distribution of deviance statistics from the permuted data sets, and the *p*-value is based on the deviance statistic when comparing models without geolocation.

For a test of hypothesis

H_0_: geolocation is unassociated with the child anemia risk for adjusting covariates (reject H0 if the percentile rank is below alpha (default alpha value = 0.05).

H_1_: geolocation is associated with child anemia risk.

In this study, *R* software with *MapGap* package is used for fitting a GAM with a two-dimensional smooth function (5–7). Typical spatial applications in the MapGAM package begin with the *predgrid()* function, which generates a regular grid of points inside the study area, potentially limited to points inside map boundaries (i.e., zonal maps imported from a shapefile or retrieved from the maps package). Then, crude or covariate-adjusted odds ratio effect estimates are generated for each grid point using the *modgam()* function to smooth by geolocation. The optimal span size, which indicates the percentage of the data included in the neighborhood for the LOESS smoother, can be determined using the *optspan()* function. The sensitivity analysis is applied with the AIC criteria for selecting the smoothing parameters in the MapGAM R packages for *modgam()* function. The optimal span sizes for the two-dimensional location predictor's LOESS smooth are chosen from a range of values between 0.05 and 0.95, with each increment of 0.05 (13, 19) for which the minimum values of AIC is the optimal span.

It is advisable to interpret the smoothed spatial terms visually. By utilizing the *modgam* plotting procedure, a call to the *colormap()* function can generate a heatmap illustrating the estimated predictions of the spatial effect. This heatmap represents the odds ratio, comparing the probabilities at each site to the median odds across all locations (51).

## 3 Results

### 3.1 Descriptive statistics for socio-demographic characteristics

**Figure 3:**
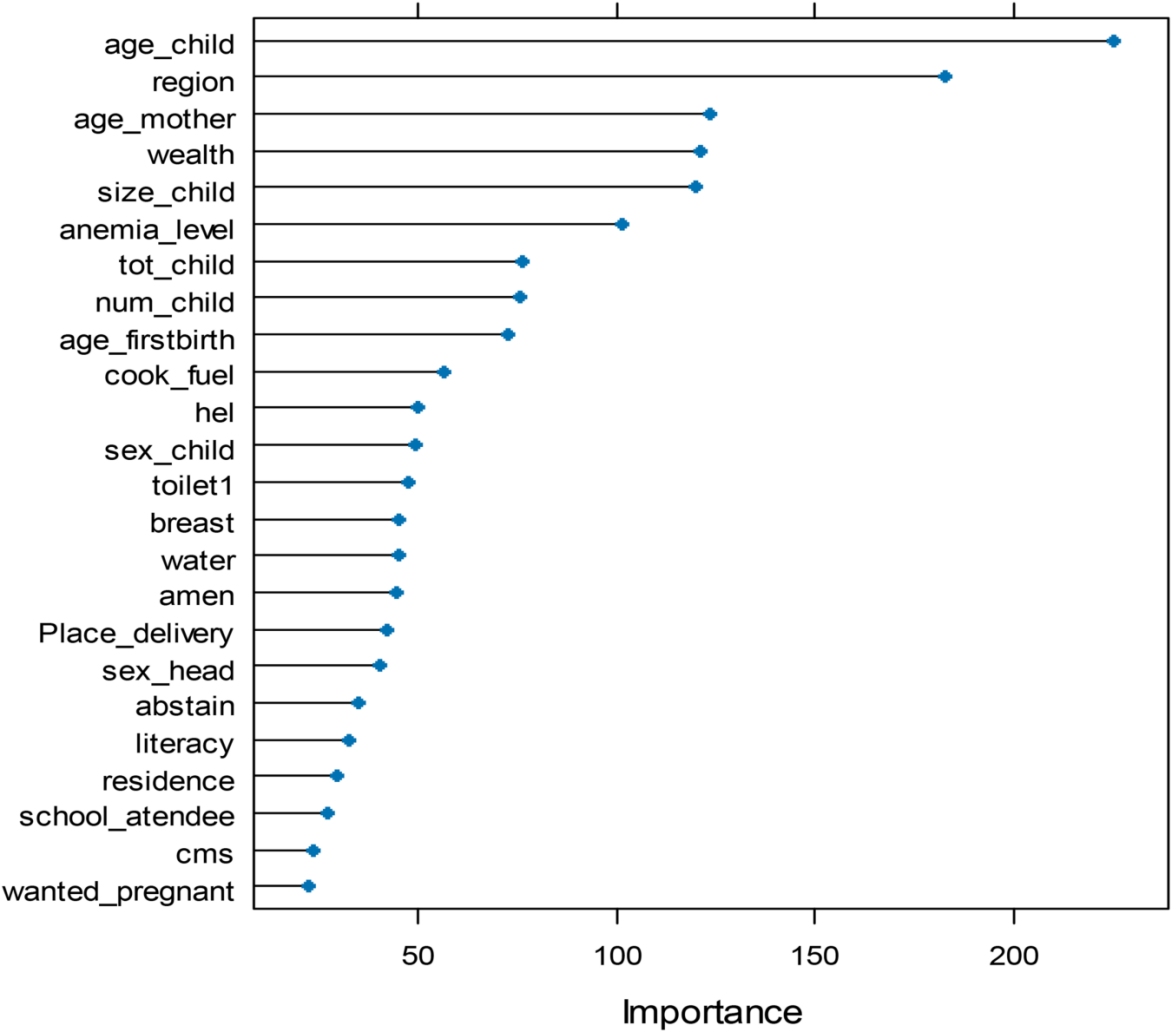
Feature importance scores based on random forest algorithm.

In this study, anemia status and associated covariates were derived from the 2016 EDHS dataset. Table 1 displays the anemia status for all potential candidate variables along with their corresponding variable importance rank values. To select the variables to our analysis we preferred at least 30% of the maximum variable importance score. This is since 30% rule of thumb is a heuristic, which keeps variables that are at least 30% important closer to the top. When looking Figure 3 and Table 1, there is a wide gap in importance scores between the variables age at first birth (72.91) and cooking fuel (56.53) and therefore we prefer variable importance greater than 67 (i.e., 67=0.3×225). Where 225 is the maximum importance score for current age of child in months.

**Table 1:** Random forest-based variable importance rankings for socioeconomic predictors of child anemia, with category frequencies and proportions.

Socio-economics characteristics	Categories	Frequency Non-anemic	Frequency anemic	Non anemic (%)	Anemic (%)	Variable importance
Age of mothers in 5-year groups	15–19	61	160	27.60	72.40	123.67
	20–24	544	906	37.52	62.48	
	25–29	896	1,374	39.47	60.53	
	30–34	701	1,009	40.99	59.01	
	35–39	527	674	43.88	56.12	
	40–44	232	248	48.33	51.67	
	45–49	85	68	55.56	44.44	
Region	Tigray	389	440	46.92	53.08	182.38
	Afar	183	561	24.60	75.40	
	Amhara	451	332	57.60	42.40	
	Oromia	426	766	35.74	64.26	
	Somalia	141	610	18.77	81.23	
	Benshangul	378	281	57.36	42.64	
	SNNPR	491	504	49.35	50.65	
	Gambella	206	298	40.87	59.13	
	Harari	125	248	33.51	66.49	
	Addis Ababa	159	146	52.13	47.87	
	Dire Dawa	97	253	27.71	72.29	
Wealth	Poorest	819	1,814	31.11	68.89	121.02
	Poorer	559	800	41.13	58.87	
	Middle	538	591	47.65	52.35	
	Richer	454	505	47.34	52.66	
	Richest	676	729	48.11	51.89	
Current age of child in months	Bellow 11	195	677	22.86	77.14	224.57
	12–23	492	1,213	38.77	71.14	
	24–37	637	1,006	47.86	61.23	
	38–47	759	827	47.86	52.14	
	48–59	963	716	57.36	42.64	
Size of child at birth	Very large	504	717	41.28	58.72	120.04
	Large	476	595	44.44	55.56	
	Average	1,338	1,816	42.42	57.58	
	Small	303	480	38.70	61.30	
	Very small	425	831	33.84	66.16	
Women's anemia level	Severe	21	91	18.75	81.25	101.74
	Mild	182	505	26.49	73.51	
	Moderate	581	1,220	32.26	67.74	
	Not anemic	2,262	2,623	46.31	53.69	
Total children ever born	1–2	936	1,275	72.33	57.67	76.49
	3–4	877	1,293	40.41	59.59	
	5–6	640	972	39.70	60.30	
	Above 7	593	899	39.75	60.25	
Maternal age at first birth	Below 15	410	638	39.12	60.88	72.91
	16–20	1,767	2,676	39.77	6.23	
	Above 21	869	1,125	43.58	56.42	
Number of living children in the household	0–2	1,035	1,415	42.24	57.76	76.16
	3–4	961	1,393	40.82	59.18	
	5–6	626	996	39.59	61.41	
	Above 7	424	635	40.04	59.96	
Type of cooking fuel	Electricity	180	144	55.56	44.44	56.53
	Charcoal	268	323	45.35	54.65	
	Wood	2,388	3,695	39.26	60.74	
	Other	210	487	43.12	56.88	
Literacy	No	2,138	3,439	38.34	61.66	32.70
	Yes	908	1,000	47.59	52.41	
Highest educational level	No education	1,858	2,979	38.41	61.59	50.51
	Primary	831	1,099	43.06	56.94	
	Secondary	230	246	48.32	51.68	
	Higher	127	115	52.48	47.52	
Place of residence	Urban	608	720	45.78	54.22	-
	Rural	2,438	3,719	39.60	60.40	
Sex of household head	Male	2,479	3,499	41.47	58.58	40.86
	Female	567	940	37.62	62.38	
Sex of child	Male	1,545	2,300	40.18	59.82	49.75
	Female	1,501	2,139	41.24	58.76	
Current marital status	Married	2,818	4,176	40.29	59.71	-
	Other	228	263	46.44	53.56	
Currently breastfeeding	No	1,215	1,489	44.93	55.07	45.56
	Yes	1,831	2,950	38.30	61.70	
Currently amenorrheic	No	1,963	2,539	43.60	56.40	44.94
	Yes	1,083	1,900	36.31	63.69	
Currently abstain from consuming alcohol	No	2,658	3,756	41.44	58.56	35.38
	Yes	388	683	36.23	63.77	
Ever attended school	No	1,858	2,979	38.41	61.59	-
	Yes	1,188	1,460	44.86	55.14	
Source of drinking water	Improved	1,446	1,830	44.14	55.85	45.48
	Unimproved	1,600	2,609	55.86	61.99	
Type of toilet facility	No	1,287	2,327	35.61	64.39	48.06
	Yes	1,759	2,112	45.44	54.56	
Place of delivery	Home	1,957	2,968	39.74	60.26	42.54
	Health center	1,089	1,471	42.54	57.46	
Wanted pregnancy	No more	188	254	42.53	57.47	-
	Yes	2,858	4,185	40.58	59.42	

Consequently, the important biological variables, current age of child in months (225), size of child at birth (120), women's anemia status (102), total number of children ever born (77), maternal age at first birth (73), and number of living children in the household (76), and the socioeconomic variables such as age of mothers in 5-year intervals (124), region (182), wealth index (121) are retained in the final model. These variables were selected for fitting the specified model (see Figure 3 and Table 1). In contrast, other covariates with low importance values were excluded from the study. The numbers in parentheses indicate the importance of scores for each variable.

We observed that the prevalence of anemia varies significantly at the regional level. Somalia has the highest rate at 81%, followed by Afar at 75% and Dire Dawa at 72% (1). In contrast, Amhara has a lower prevalence of 42%, followed by Benishangul at 43% and Addis Ababa at 48%. Economic status also plays a crucial role in anemia risk. Children from the poorest families are at the highest risk of anemia, followed by those from poorer families. Additionally, children aged 6–11 months have the highest risk of anemia, while those aged 48–59 months have a lower risk. Furthermore, the anemia risk in children is significantly associated with the severe anemic status of their mothers, who exhibit the highest rates of anemia compared to mothers with less severe anemia.

### 3.2 Estimating and mapping spatial effects adjusted for covariates

GAMs were employed to smooth the covariates, specifically focusing on two-dimensional predictors associated with geolocations while also adjusting for covariates. The crude and adjusted for covariates analysis statistics test values are presented in Table 2. According to the crude analysis, the prevalence of childhood anemia had a significant association with the respondents’ spatial locations (longitude, latitude), as indicated by the *p*-value (0.000) used to evaluate the global spatial effect. Geographic variations were statistically significant prior to the variables being adjusted for geographical geolocations. According to the findings in Table 2, the ideal span size that reduced the AIC was 0.15, by selecting by sensitivity analysis, meaning that 15% of the nearby dataset was utilized to smooth the geolocation parameters. Considering that the *p*-value to assess the global spatial effect of children anemia is 0.000, the findings showed that, after controlling for variables, there were significantly significant spatial differences between location and childhood anemia prevalence (Table 2).

**Table 2:** Optimal span size selection for crude and covariate adjusted spatial effects.

Parameters	Unadjusted (crude)	Adjusted for covariates
Deviance statistic	9,431.596	8,698.54
AIC	9,513.686	8,862.98
*p*-value	0.000	0.000
Span size	0.15	0.15

Estimating and mapping the spatial distribution of anemia risk is crucial for identifying health disparities. Epidemiologists are particularly interested in creating risk surfaces that account for individual-level confounding variables (52). The adjusted odds ratios (AOR) and their corresponding *p*-values, which reflect potential covariate-adjusted spatial effects using GAMs, are detailed in Table 3.

**Table 3:** Adjusted covariate spatial effects odds ratios for socioeconomic variables using a generalized additive model.

Variables	Categories	AOR	*p* value
Age of mothers in 5-year groups	1 = “15–19” (ref.)	-	-
	2 = 20–24	0.90	0.536
	3 = 25–29	0.93	0.682
	4 = 30–34	0.88	0.524
	5 = 35–39	0.84	0.394
	6 = 40–44	0.77	0.259
	7 = 45–49	0.62	0.082
Region	1 = Tigray (ref.)	-	-
	2 = Afar	1.66	<0.000
	3 = Amhara	0.80	0.068
	4 = Oromia	1.14	0.049
	5 = Somalia	1.10	0.759
	6 = Benishangul	0.65	0.009
	7 = SNNPR	0.66	0.055
	8 = Gambella	1.63	0.032
	9 = Harari	0.27	<0.000
	10 = Addis Ababa	0.85	0.427
	11 = Dire Dawa	1.74	0.006
Wealth index	1 = Poorest (ref.)	-	-
	2 = Poorer	0.86	0.059
	3 = Middle	0.69	<0.000
	4 = Richer	0.72	<0.000
	5 = Richest	0.50	<0.000
Current age of child in months	0 = 6–11 (ref.)	-	-
	1 = 12–23	0.73	0.0015
	2 = 24–37	0.41	<0.000
	3 = 38–47	0.27	<0.000
	4 = “48–59”	0.18	<0.000
Size of child at birth	1 = Very large (ref)	-	-
	2 = Larger than average	0.93	0.400
	3 = Average	1.03	0.788
	4 = Smaller than average	1.13	0.266
	5 = Very small	1.14	0.171
Women's anemia level	1 = Severe (ref)	-	-
	2 = Mild	0.69	0.147
	3 = Moderate	0.72	0.228
	4 = Not anemic	0.50	0.008
Total children ever born	0 = 1–2 (ref)	-	-
	1 = 3–4	1.10	0.551
	2 = 5–6	0.92	0.707
	3 = “>=7”	1.01	0.978
Maternal age at first birth	1 = Below 15 (ref.)	-	-
	2 = 16–20	0.97	0.644
	3 = Above 21	0.91	0.333
Number of living children in the household	0 = 1–2 (ref.)	-	-
	1 = 3–4	1.10	0.061
	2 = 5–6	1.33	0.014
	3 = “>7”	1.15	0.054

Children residing in the Afar region are 1.66 times more likely to develop childhood anemia (AOR = 1.66, *p*-value = 0.000) than those in the Tigray region. Similarly, children in Oromia have a 1.14 times higher likelihood (AOR = 1.14, *p*-value = 0.049), those in Gambella are 1.63 times more likely (AOR = 1.63, *p*-value = 0.032), and children in Dire Dawa are 1.74 times more likely (AOR = 1.74, *p*-value = 0.006) to develop anemia when compared to their counterparts in Tigray. In contrast, children living in Benishangul are statistically significantly less likely to develop anemia (AOR = 0.65, *p*-value = 0.009). At the same time, those in Harari show an even lower likelihood (AOR = 0.27, *p*-value = 0.000) compared to children in Tigray.

Children aged 12–23 months (AOR = 0.73, *p*-value = 0.0015), 24–37 months (AOR = 0.41, *p*-value < 0.0001), 38–47 months (AOR = 0.27, *p*-value < 0.0001), and 48–59 months (AOR = 0.18, *p*-value < 0.0001) were statistically significantly less likely to develop anemia when compared to those aged 6–11 months.

Additionally, the likelihood of developing anemia decreased by 31% (AOR = 0.69, *p*-value < 0.0001) for children from families with a middle wealth index, by 28% (AOR = 0.72, *p*-value < 0.0001) for those from richer families, and by 50% (AOR = 0.50, *p*-value < 0.0001) for children from the wealthiest families, in comparison to those from the poorest families.

Moreover, children of non-anemic mothers had a 50% lower risk of developing anemia (AOR = 0.50, *p*-value = 0.008) compared to children of mothers with severe anemia. Finally, children living in households with 5–6 siblings were 1.33 times more likely to develop anemia compared to those with only 1–2 siblings.

**Figure 4:**
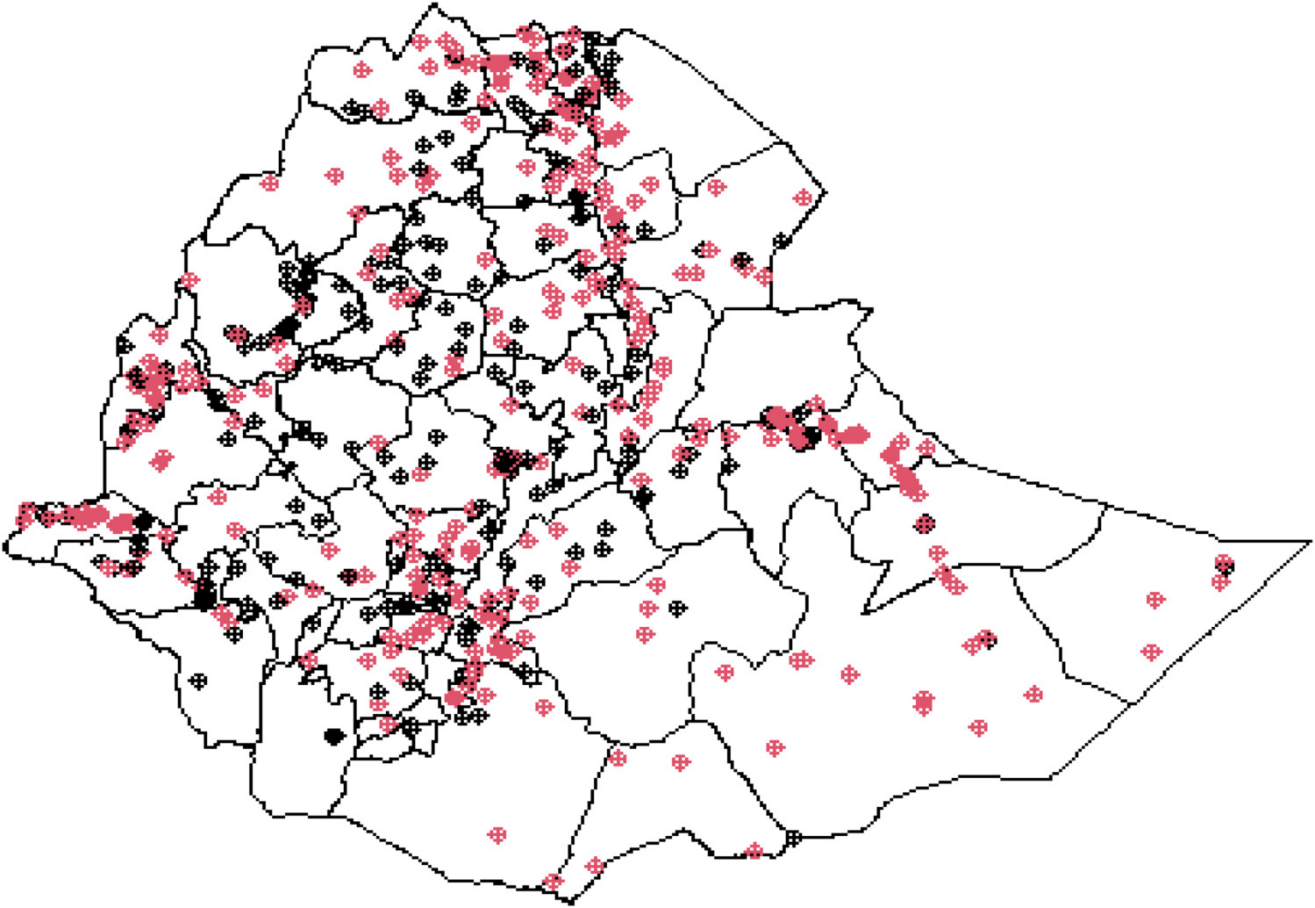
Geolocation of child anemia observations across the local zones in Ethiopia.

The Ethiopian zonal maps illustrating the locations where survey datasets were collected, based on the 2016 EDHS, are shown in Figure 4. This map presents the geolocations of child anemia observations across various administrative zones in Ethiopia. Anemic cases are marked in red on the map, while non-anemic cases are indicated in black.

**Figure 5:**
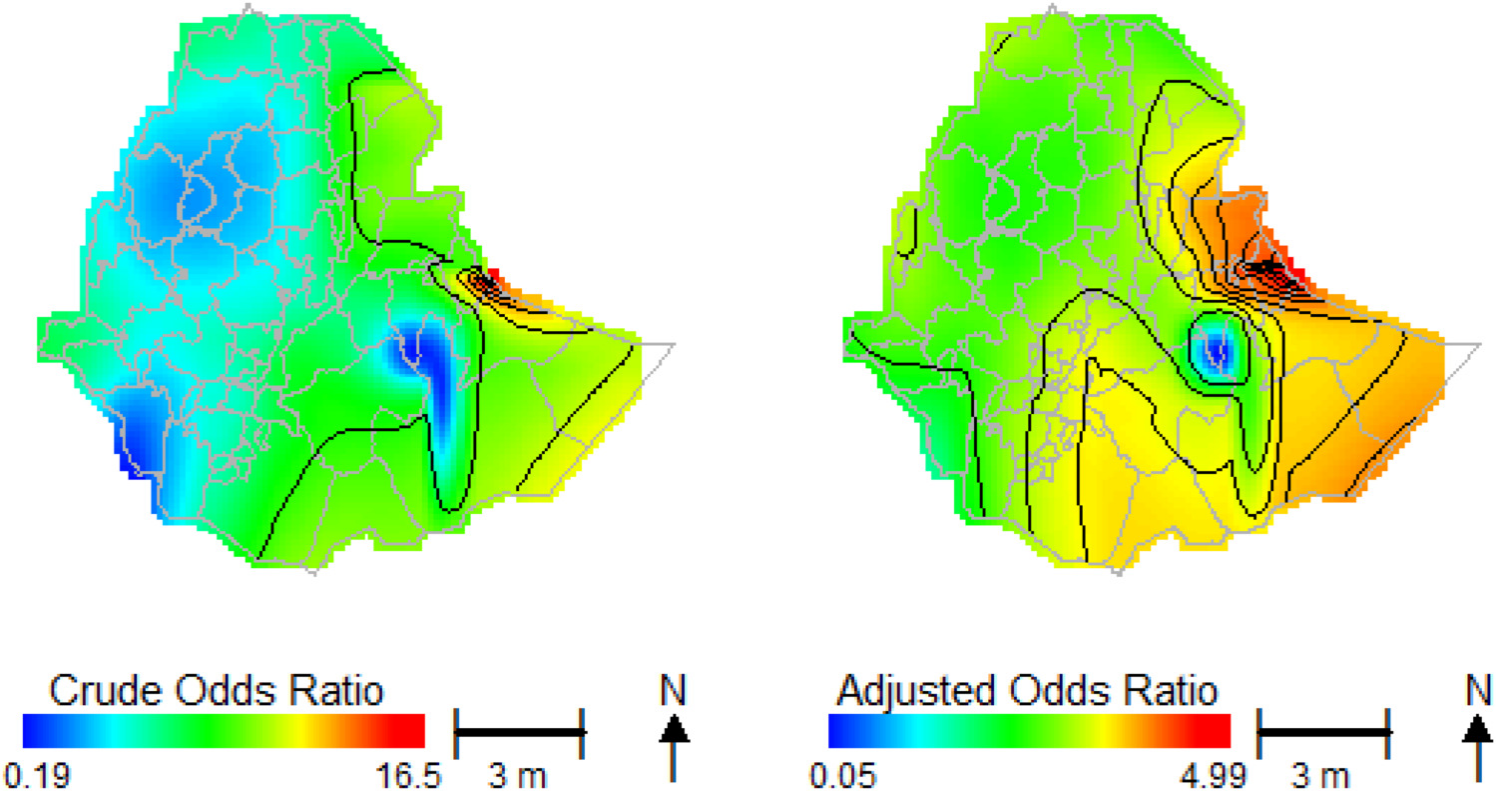
The crude and adjusted odds ratio of spatial effect predictions.

The connection between spatial locations and the risk of childhood anemia is depicted in Figure 5, which features a heatmap illustrating both crude and covariate-adjusted spatial effects. The crude odds ratio map (Figure 5, left) is based exclusively on two spatial characteristics (geolocation) that define a geographic smoothing term related to the risk of child anemia. In contrast, the adjusted odds ratio map (Figure 5, right) accounts for all covariate data to provide a more comprehensive estimate of the spatial impacts.

Evaluating the smoothed spatial terms visually, as shown in Figure 5, is particularly effective. The heatmap represents the spatial disparities in anemia across Ethiopian administrative zones, displaying the estimated spatial effect predictions. This visualization compares the odds ratio for each location against the median odds across all locations. In Figure 5, regions with a higher likelihood of child anemia are indicated in red, while those with a lower likelihood are represented in blue.

**Figure 6:**
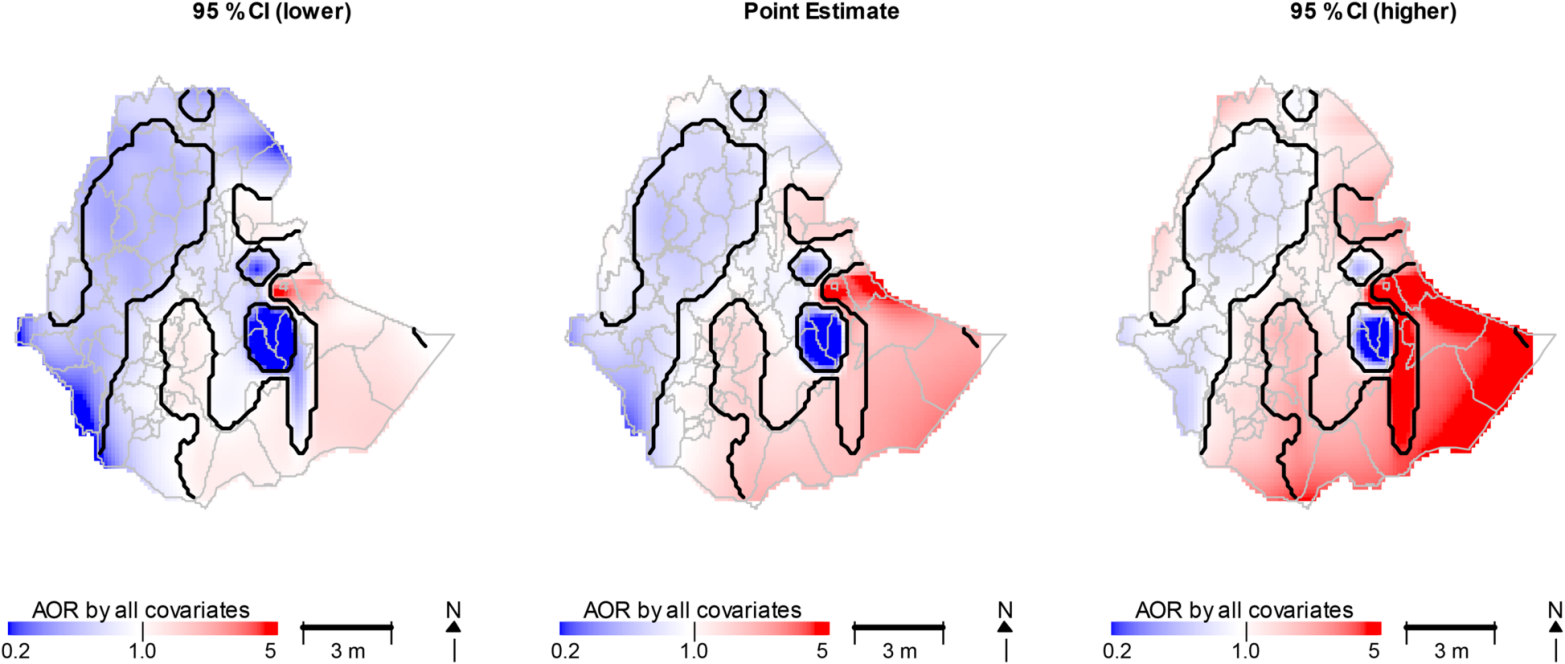
The confidence interval and point estimates of AOR with all covariates adjusted spatial effects.

MapGAM provides point estimates for each spatial location, along with point-wise standard errors and confidence intervals. Significant regions are identified using the 95% probability intervals of the non-parametric smoothing effects, as illustrated in Figure 6. To visualize the inference for spatial effects, the “plot” function displays all point estimates alongside their corresponding lower and upper confidence interval bounds. Areas where the confidence intervals do not include 0 (on the log estimated effect scale) can be represented on the map by plotting contours of an indicator vector. This vector indicates whether 0 is positioned below, within, or above the confidence intervals at the grid points.

Table 4 presents the summary statistics for spatial effect predictions, including the residual deviance and the AIC for potential predictor variables contributing to anemia prevalence. The results include the minimum AIC (where a smaller value indicates a better fit), the residual deviance, and the spatial prediction effects.

**Table 4:** Residual deviance, AIC, and summary statistics for covariate-adjusted spatial effect predictions of anemia by socioeconomic variables.

Socio-economics characteristics	Residual deviance	AIC	Spatial effect predictions				
			Min	Q1	Mean	Q3	Max
Age of mothers in 5-year groups	9,404	9,498	−4.20	−0.54	0.10	0.81	2.56
Region	9,363	9,465	−2.40	−0.36	0.22	0.85	2.64
Wealth	9,340	9,431	−5.68	−0.55	0.02		
Current age of child in months	8,942	9,032	−4.24	−0.58	0.11	0.89	2.81
Size of child at birth	9,411	9,502	−4.16	−0.55	0.08	0.81	2.60
Women's anemia level	9,373	9,463	−3.68	−0.52	0.06	0.70	2.37
Total children ever born	9,431	9,519	−3.78	−0.56	0.09	0.82	2.56
Age of respondent at first birth	9,427	9,514	−3.90	−0.56	0.09	0.80	2.55
Number of living children	9,428	9,516	−3.76	−0.56	0.09	0.82	2.55

**Figure 7:**
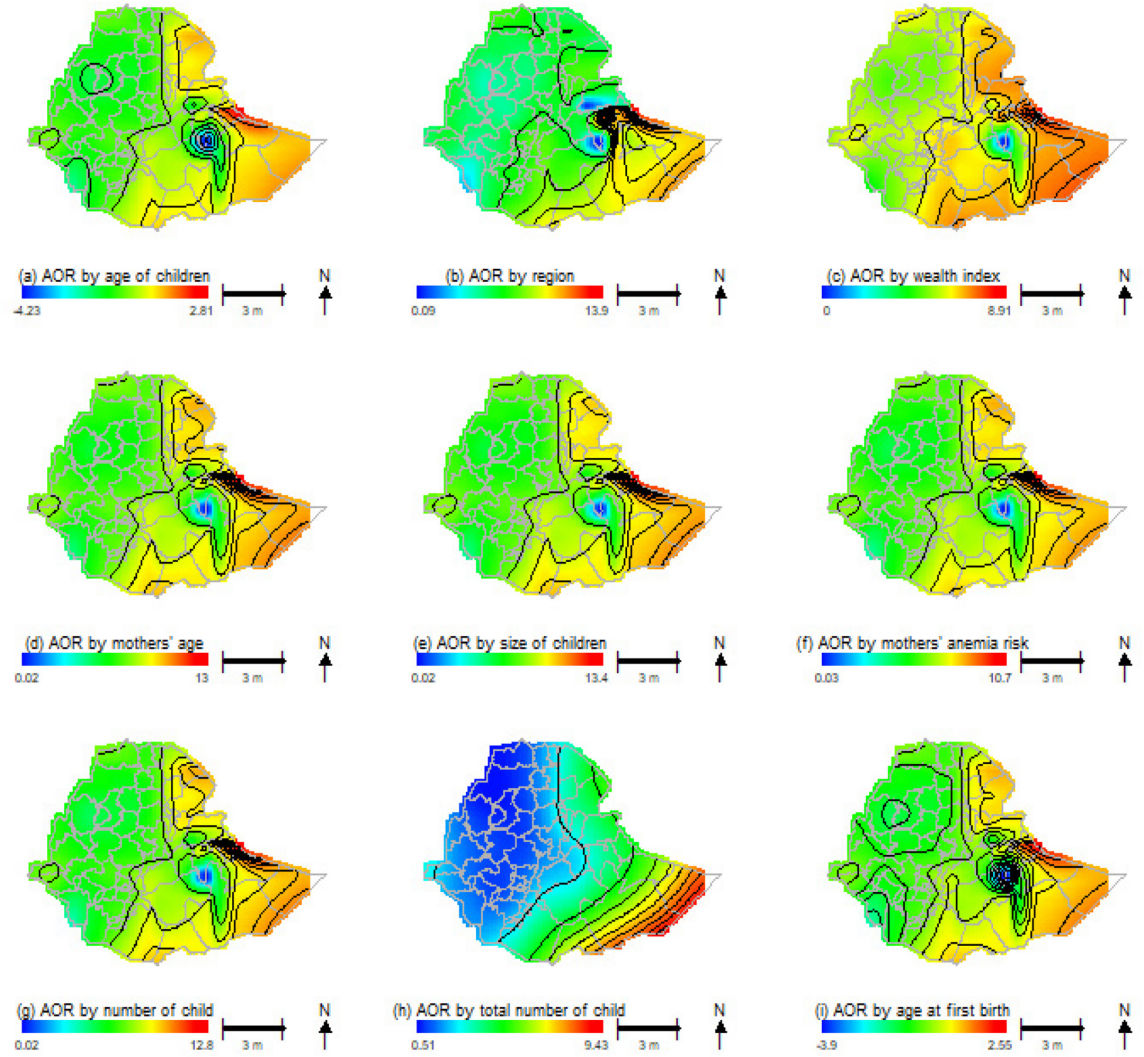
Nine maps display the Adjusted Odds Ratio (AOR) in Ethiopia by various factors: **(a)** children's age, **(b)** region, **(c)** wealth index, **(d)** mothers' age, **(e)** size of children, **(f)** mothers' anemia risk, **(g)** number of children, **(h)** total number of children, and **(i)** age at first birth. Each map uses a color gradient to represent AOR values, with a scale bar and north arrow for reference.

The potential contribution of each variable to the risk of child anemia, adjusted for geolocation (latitude and longitude) in each zone, is displayed in Figure 7. This figure illustrates the contributions of various risk predictor variables to the geographic patterns of anemia odds ratios for children aged 6–59 months while controlling for other factors.

In the figure, key predictor variables including the child's current age in months, region, wealth index, maternal age, birth size of children, maternal anemia risk, number of children in the household, maternal age at first birth, and total number of children born show differing effects on anemia prevalence across various zones in Ethiopia when compared to their reference groups. Some regions exhibit a positive impact from these predictor variables, while others show negative effects.

The regions with the highest risk of child anemia are in the Northeastern, Eastern, and Southeastern parts of the country. In contrast, areas with the lowest risk can be found in the Northwestern, Western, and Southwestern regions. For instance, when factors such as wealth index, maternal anemia levels, and the age of the child are considered, the odds ratio for childhood anemia is considerably elevated in the Northeastern, Eastern, and Southeastern areas. On the other hand, the odds ratio for childhood anemia is significantly reduced in the Northwestern, Western, and central regions of the country.

## 4 Discussion

The purpose of this study is to consider the spatial inequalities and factors that influence childhood anemia in Ethiopian children aged 6–59 months from various zones. Though there were studies in spatial distributions of anemia prevalence at the regional and national levels (24, 34, 35), we are aware of that there was limited investigations explored on the spatial disparities across the zones using individual level data for smoothing the geolocation variables and adjusting linearly for confounding variables. Accordingly, the current study involved in mapping and visualizing the spatial effects adjusted for covariates, explicitly focusing on the risk of child anemia within the various administrative zones of Ethiopia. In contrast to other studies (24, 34, 35), the current analysis was mapped individual-level odds ratios across the zones, the third administration layer of the country. The odds ratios of the potential risk factors were estimated in relation to the zonal level geographical distributions of the prevalence of anemia among children aged 6–59 months. The odds ratios for the individual level variables, as indicated in Figure 7, linked to the geolocations were plotted in the administrative zones of Ethiopia (53, 54).

This study was in line with previous findings from HIV data conducted in South Africa and Ethiopia (52, 55). However, research in low- and middle-income nations were mapping the prevalence of anemia in those nations (56–58). Furthermore, other research on the prevalence of anemia in Ethiopia has mapped it across the second administration level (59). However, this methodology enabled spatially adjust the odds of individual variables to assess their spatial effects on the risk of anemia. Our findings are also consistent with research conducted in sub-Saharan Africa (60) providing additional validation for our conclusions.

The geospatial analysis reveals that most anemia hotspot areas were in zones of Somali, Afar, and Dire Dawa city administration, whereas the lowest anemia hotspots were primarily observed in zones of Amhara, Benishangul, and Addis Ababa. Anemia prevalence exhibited considerable variation among local administrations zones of Ethiopia. In the Afar region, the zones identified as high hotspots for anemia include Zone 1, Zone 5, and Shinile. Furthermore, Jijiga, East Hararge, Degahabur, Welewel, Shabelle, Liben, and Bale zones in Somalia region ranked among the highest hotspot areas. This was since these zones were primarily inhabited by pastoralist communities, experience seasonal mobility for livelihood, were frequently affected by drought, and were geographically remote, which limited access to transportation and health services. These indicated that, in comparison to other places, pastoralist communities are at a higher risk of anemia. These were due to different factors such as food insecurity, lower economic status, and nutritional deficiency (61, 62). In contrast, most zones in Amhara and Tigray regions were classified as low hotspot areas.

The observed regional and zonal variations may be attributed to differences in dietary habits, the distribution of infectious diseases, and the accessibility of maternal and child health care services (63). The socio-economic status of a household was significantly linked to the prevalence of anemia among children under 5 years of age. Children from households in the poorest and poorer wealth quantiles faced a higher risk of anemia, with probabilities exceeding 68.8% and 58.87%, respectively, when compared to their peers in the wealthiest quantile. This finding aligns with studies conducted in Ethiopia (25, 64–67), Sub-Saharan Africa (68), Nigeria (69), and Brazil (70, 71). This association may be attributed to the fact that children from wealthier households were more likely to have access to a balanced diet rich in macro and micronutrients, including essential vitamins and minerals, and appropriate medical care.

Research studies indicated that children from lower socioeconomic backgrounds were more susceptible to easily preventable diseases and various nutritional deficiencies, including anemia (61, 62, 72, 73). This was a consequence of the limited dietary variety often seen in rural households, which struggle with inadequate resources to provide balanced meals (23). In this study, over 80% of participants were from rural areas (74–78). Consequently, children from the poorest families were less likely to have access to a balanced diet, as they cannot afford or utilize a range of foods. This lack of dietary diversity increased their risk of poor health conditions, including anemia caused by factors such as parasitic infections.

Children aged 6–11 months were more likely to develop anemia compared to those older than 12 months. Studies conducted in Ethiopia (59, 79–81), Uganda (82), Sub-Saharan Africa (75), Togo (83), Bangladesh (74, 84), Brazil (85), Asia and India (86), Burma (71), Sydney (87), and Nepal (88) support this conclusion. One reason for this increased risk is that younger children had a higher need for micronutrients as they grow (72, 89). If they did not receive these essential nutrients, they may become anemic (72). Another explanation is that older children tend to have a more diversified diet, which often includes sufficient iron-rich foods, helping to prevent anemia (74, 90). Children over the age of one typically consumed a variety of iron-rich foods such as cereals, meats, poultry, and fish (91, 92). Moreover, nutritional issues were more prevalent in younger children than in older ones. Younger children, especially those living in unhygienic environments were also more vulnerable to infectious diseases like intestinal helminthes, as they may put contaminated objects in their mouths (93–95).

Children from households with more than two family members were more likely to be anemic compared to children from households with two or fewer family members. This finding was supported by research from Ethiopia (67, 79, 96, 97), Uganda (82), Sub-Saharan Africa (SSA) (75), Brazil (70, 98), India (99), Switzerland (90), and Burma (71). The higher incidence of anemia in larger families may be attributed to several factors, including increased transmission of communicable diseases, inadequate nutrient intake, and competition for food. Additionally, larger families may face challenges in accessing appropriate health care services, which can lead to infections and nutritional deficiencies (74, 100). These issues can worsen the quality of care for children and increase their risk of developing anemia (76).

Maternal anemia was significantly linked to the onset of childhood anemia, a correlation supported by studies conducted in Ethiopia (97), Togo (83), Cuba (101), Burma (71), Brazil (85, 98), Kuwait (102), and Nepal (88). This relationship can be attributed to children often sharing common environmental, socioeconomic, and dietary conditions with their mothers (103). Furthermore, inadequate maternal iron reserves during pregnancy and lactation can adversely affect the iron levels in their children (86, 99, 104). Additionally, maternal anemia increases the risks of low birth weight, premature delivery, and maternal mortality (105), all of which contribute to a higher likelihood of childhood anemia.

### 4.1 Limitations

EDHS collects data in every 5 years, however, due to national instability and the COVID-19 pandemic's effects, data was not gathered in 2021 after the 2016 round, and no further polls have been carried out. In 2011, the World Health Assembly (WHA) established goals to cut the risk of anemia in half by 2025. The Ethiopian government strives to meet the WHA targets, however we are uncertain if this has been accomplished or not because the data was not timely collected. In fact, there will be an expected discrepancies of the anemia prevalence within this 9 years gap. However, academicians, stakeholders, and national policy makers continue to use the 2016 EDHS data to inform policy decisions for the government's legislative body.

Geo-masking or geo-privacy might be impacted the estimations of the spatial effect by introducing bias and inaccuracy, which could result in incorrect interpretations of the spatial pattern or relationship. In this study, the latitude and longitude of survey clusters are moved by 0–2 km for urban regions and 0–5 km for rural areas. The focus of this study is spatial effects estimates at bigger area levels (zones and regions), therefore moving individuals’ location for privacy within certain distance might have an negative impact on the analysis.

## 5 Conclusion

This study found that children anemia in Ethiopia is highly influenced by both individual-level factors such as age of the child, age of the mother, wealth index, mother's anemia risk, age of first birth, and number of children in the family, and spatial determinants, notably in pastoralist zones in Afar and Somalia. These findings underscore the importance of using spatial models to uncover hidden zonal vulnerabilities. The significant geographical variation in childhood anemia risk over zones differed based on the spatially adjusted covariates. Notably, hotspot areas were predominantly located within pastoralist communities, especially in several zones of Ethiopia's Afar and Somali regions. Regional differences in anemia risk were shaped by factors such as maternal health, wealth index, and age of child, which varied across zones. These findings will aid policymakers in developing geographically targeted strategies to address and mitigate anemia risks effectively across the Ethiopian local level administrations.

## Data Availability

Publicly available datasets were analyzed in this study. This data can be found here: https://www.dhsprogram.com.
