# Supplementary material for: Mapping the covariate-adjusted spatial effects of childhood anemia in Ethiopia using a semi-parametric additive model
Source: Front Pediatr. 2025 Aug 21;13:1559140. doi: 10.3389/fped.2025.1559140 (PMC12408295; doi:10.3389/fped.2025.1559140)
Supplement: Supplementary file 1 [file Datasheet1.pdf]

Generalized Additive Model (GAM) with Decomposed Spatial Effect

Let Y_i be a response variable following an exponential family distribution:

$$p_Y(y_i; \theta_i, \phi) = \exp\left(\frac{y_i\theta_i - b(\theta_i)}{a(\phi)} + c(y_i, \phi)\right), \quad i = 1, 2, \ldots, N$$

The expected value of Y_i is:

$$\mu_i = \mathbb{E}[Y_i], \quad \text{and} \quad g(\mu_i) = \eta_i$$

Where:

- $g(\cdot)$ is a known link function,
- η_i is the additive predictor,
- y_i: observed response for the i-th unit,
- θ_i: natural (canonical) parameter for the i-th unit — typically a function of μ_i,
- ϕ: dispersion (or scale) parameter — controls variability across all units,
- $a(\phi)$: a known function of ϕ, often $a(\phi) = \phi$,
- $b(\theta_i)$: cumulant or log-partition function,
- $c(y_i, \phi)$: normalization function to ensure the density integrates to 1.

GAM Structure

In a Generalized Additive Model (GAM), the predictor η_i is given by:

$$\eta_i = \beta_0 + \mathbf{x}_i^T \boldsymbol{\beta} + f(u_i, v_i)$$

- β_0 is the intercept,
- $\mathbf{x}_i \in \mathbb{R}^p$ is a vector of covariates,
- $\boldsymbol{\beta} \in \mathbb{R}^p$ is the vector of coefficients,
- $f(u_i, v_i)$ is a spatial effect modeled over coordinates (u_i, v_i).

Decomposition of the Spatial Effect

To separate linear (parametric) spatial trends from nonlinear (smooth) effects, the spatial function $f(u_i, v_i)$ is decomposed as:

$$f(u_i, v_i) = \gamma_u u_i + \gamma_v v_i + s(u_i, v_i)$$

- $\gamma_u u_i + \gamma_v v_i$ is the **parametric linear trend** in space,
- $s(u_i, v_i)$ is the **nonparametric smooth function** capturing spatial variation not explained by the linear trend.

Redefined GAM Model

Substituting the decomposition into the GAM predictor, we get:

$$\eta_i = \beta_0 + \mathbf{x}_i^T \boldsymbol{\beta} + \gamma_1 u_i + \gamma_2 v_i + s(u_i, v_i)$$

Therefore, the full model becomes:

$$g(\mu_i) = \beta_0 + \sum_{j=1}^{p} x_{ij}\beta_j + \gamma_1 u_i + \gamma_2 v_i + s(u_i, v_i)$$

Interpretation

- The terms $\gamma_1 u_i + \gamma_2 v_i$ model large-scale spatial trends (e.g., elevation, latitude effects),
- The smooth term $s(u_i, v_i)$ captures local, nonlinear spatial variations (e.g., clustering),
- The combination enhances interpretability and helps avoid overfitting.

Estimation

The log-likelihood for all N observations is:

$$\ell(\theta, \phi) = \sum_{i=1}^{N} \left[\frac{y_i\theta_i - b(\theta_i)}{a(\phi)} + c(y_i, \phi) \right]$$

Let us denote this log-likelihood as:

$$\log L(\beta, \gamma, s)$$

since θ_i is determined by the linear predictor η_i, which depends on β, γ, s.
Where,

- Parametric covariates $\boldsymbol{\beta}$,

- Linear spatial coefficients $\boldsymbol{\gamma}$ (e.g., γ_1, γ_2),
- A smooth spatial function $s(u, v)$.

The model is typically estimated via penalized likelihood methods:

$$\text{PL} = \log L(\beta, \gamma, s) - \lambda \int \left[\left(\frac{\partial^2 s}{\partial u^2} \right)^2 + 2 \left(\frac{\partial^2 s}{\partial u \partial v} \right)^2 + \left(\frac{\partial^2 s}{\partial v^2} \right)^2 \right] du \, dv$$

where λ is a smoothing parameter controlling the trade-off between model fit and smoothness of $s(u, v)$.

Component-by-Component Breakdown

1. Log-Likelihood Term: $\log L(\boldsymbol{\beta}, \boldsymbol{\gamma}, s)$

This is the standard likelihood function used in generalized linear models (GLMs), based on the assumed distribution of Y_i (e.g., Binomial). It measures how well the model fits the observed data.

2. Penalty Term

$$\int \left[\left(\frac{\partial^2 s}{\partial u^2} \right)^2 + 2 \left(\frac{\partial^2 s}{\partial u \partial v} \right)^2 + \left(\frac{\partial^2 s}{\partial v^2} \right)^2 \right] du \, dv$$

This is a *roughness penalty* that discourages overfitting by penalizing the wiggliness of the spatial surface $s(u, v)$. The components represent:

- $\left(\frac{\partial^2 s}{\partial u^2} \right)^2$: curvature in the east-west direction,
- $\left(\frac{\partial^2 s}{\partial v^2} \right)^2$: curvature in the north-south direction,
- $\left(\frac{\partial^2 s}{\partial u \partial v} \right)^2$: diagonal interaction curvature.

3. Smoothing Parameter λ

The smoothing parameter λ controls the trade-off between:

- **Model fit** (when λ is small): allows more flexibility in $s(u, v)$, but risks overfitting,
- **Smoothness** (when λ is large): enforces smoother surfaces, but may underfit if too restrictive.

Common methods for selecting λ include:

- Cross-validation,
- Generalized Cross-Validation (GCV),
